# Impact of the COVID-19 pandemic on mental health and service use of people with severe mental illness

**DOI:** 10.1192/j.eurpsy.2022.1352

**Published:** 2022-09-01

**Authors:** A. Mueller-Stierlin, F. Meixner, J. Lehle, R. Kilian

**Affiliations:** Ulm University, Department Of Psychiatry Ii, Guenzburg, Germany

## Abstract

**Introduction:**

The COVID-19 pandemic has a huge impact on the provision of mental health care. Particularly the limitations of face-to-face contacts and the access to treatment facilities can be expected to have significant negative effects on the practice of psychiatric treatment and outcomes. To date the extent and the severity of these effects in people with severe mental illnesses are rarely investigated in Germany.

**Objectives:**

We investigated the impact of the COVID-19 pandemic on mental health and service use of people with severe mental illness in Germany.

**Methods:**

As part of a pragmatic randomized trial on the effectiveness of an integrated community mental health care program that started immediately after the first COVID-19 wave in June 2020, 1000 people with severe mental illness from different regions in Germany have been asked for the effects of the COVID-19 pandemic on their mental health care and on their general living conditions. Multivariate regression models were computed to estimate the effects of the patients’ COVID-19 experiences on the outcome parameters empowerment (EPAS), psychosocial impairment (HoNOS) and unmet needs (CAN).

**Results:**

Using prospective data in a large sample of people with mental illness, we will be able to examine the extent to which the pandemic has affected participants’ mental health, their social lives, but also the use of mental health care services.

**Conclusions:**

The data will help to examine the impact of the pandemic on people with severe mental illness in a comprehensive way and will provide evidence where immediate action is needed to reduce further burdens and inequities.

**Disclosure:**

No significant relationships.

